# Potential of the zebrafish (*Danio rerio*) embryo test to discriminate between chemicals of similar molecular structure—a study with valproic acid and 14 of its analogues

**DOI:** 10.1007/s00204-022-03340-z

**Published:** 2022-08-03

**Authors:** Katharina Brotzmann, Sylvia E. Escher, Paul Walker, Thomas Braunbeck

**Affiliations:** 1 Aquatic Ecology and Toxicology Group, Center for Organismal Studies, University of Heidelberg, Im Neuenheimer Feld 504, 69120 Heidelberg, Germany; 2 Fraunhofer Institute for Toxicology and Experimental Medicine (ITEM), Nikolai-Fuchs-Strasse 1, 30625 Hannover, Germany; 3 Cyprotex Discovery, No. 24 Mereside, Alderley Park, Nether Alderley, Cheshire, SK10 4TG UK

**Keywords:** Zebrafish embryos, Liver toxicity, Structure–activity relationship (SAR), Valproic acid, Discrimination of analogues

## Abstract

Valproic acid is a frequently used antiepileptic drug and known pediatric hepatotoxic agent. In search of pharmaceuticals with increased effectiveness and reduced toxicity, analogue chemicals came into focus. So far, toxicity and teratogenicity data of drugs and metabolites have usually been collected from mammalian model systems such as mice and rats. However, in an attempt to reduce mammalian testing while maintaining the reliability of toxicity testing of new industrial chemicals and drugs, alternative test methods are being developed. To this end, the potential of the zebrafish (*Danio rerio*) embryo to discriminate between valproic acid and 14 analogues was investigated by exposing zebrafish embryos for 120 h post fertilization in the extended version of the fish embryo acute toxicity test (FET; OECD TG 236), and analyzing liver histology to evaluate the correlation of liver effects and the molecular structure of each compound. Although histological evaluation of zebrafish liver did not identify steatosis as the prominent adverse effect typical in human and mice, the structure–activity relationship (SAR) derived was comparable not only to human HepG2 cells, but also to available in vivo mouse and rat data. Thus, there is evidence that zebrafish embryos might serve as a tool to bridge the gap between subcellular, cell-based systems and vertebrate models.

## Introduction

In an effort to reduce animal tests while maintaining the security of new industrial chemicals, pesticides, biocides, cosmetics, pharmaceuticals and drugs, development and implementation of alternative and other new approach methods advanced within the last years, driven by modern legislations for chemical control as REACH (Registration, Evaluation, Authorization and Restriction of Chemicals) (EU 2006), the EU Cosmetics Regulation (EU 2009) and the current EU Animal Welfare Regulation (EFSA 2021; EMA 2021; EU 2010). Projects such as the European in vitro “flagship” toxicology project EU-ToxRisk investigate new alternative-to-animal approaches to chemical safety evaluation (Daneshian et al. 2016; Escher et al. 2019; Leist et al. 2017) and also address alternatives to mammalian teratogenicity testing of metabolites and analogues. Especially in search of pharmaceuticals with a higher efficiency and/or reduced hazard for adverse effects in humans, the toxicological profiles of metabolites and analogues of an original drug are of major interest. Therefore, the capacity of a method to discriminate between chemicals of similar molecular structure has become a mandatory characteristic of any new approach methodology (NAM). Studies by Löscher and Nau (1985) as well as Nau et al. (1991) already documented this ability for the mouse model by investigating the anticonvulsant and neural tube defect-inducing potency of valproic acid and a set of its analogues, and deriving structure-teratogenicity relationships for these observations. Following the current trend to substitute mammalian test systems by alternative methods, “lower” vertebrates have come into focus and have been challenged with respect to their potential also to discriminate between chemicals of similar molecular structure, and whether these capacities might be used for the toxicological evaluation of new compounds.

To investigate this issue, the zebrafish (*Danio rerio*) embryo was chosen for its high concordance of at least 80% to mammalian developmental toxicity (Bachmann 2002; Brannen et al. 2010; Nagel 2002) or rodent models, and even humans (MacRae and Peterson 2015; Postlethwait et al. 2000). Originally designed as an alternative model system to acute fish toxicity tests in ecotoxicology such as OECD TG 203 (OECD 2019), the fish embryo acute toxicity test (FET) (Braunbeck et al. 2015; OECD 2013) soon received increasing attention from the toxicological and pharmaceutical sectors as well. OECD TG 236 is based on the early non-feeding developmental stages of fish, which are not regarded protected according to current EU animal welfare legislation (EU 2010; Strähle et al. 2012), and, as a small cyprinid, zebrafish is not only inexpensive, easy to maintain and to breed in large numbers, but also provides fully transparent embryos, which allow continuous access to developmental disorders in a model whole organism system outside the (e.g., mammalian) mother (Braunbeck et al. 2015; Quevedo et al. 2018). Furthermore, genetic investigations of these small organism revealed high association with human diseases of approximately 84%, and a large number of drug metabolism pathways shared by humans and zebrafish (Howe et al. 2013; MacRae and Peterson 2015; Uechi and Kenmochi 2019). Many pathways identified in human found a zebrafish counterpart (Howe et al. 2013; MacRae and Peterson 2015; Uechi and Kenmochi 2019), and about 70% of human genes have at least one obvious zebrafish orthologue (Howe et al. 2013). Moreover, teratogenic types of effects recorded in zebrafish could frequently be correlated with corresponding observations in mammals, which indicates the utility and efficiency of the zebrafish embryo model for the detection of at least strong mammalian teratogenic compounds and toxicants (Ball et al. 2014; Brannen et al. 2010; Iida et al. 2019; Kim et al. 2011). Thus, over the last two decades, the zebrafish embryo has become a well-studied tool for the investigation of general vertebrate development and diseases, as well as one of the most promising models not only in ecotoxicity testing, but also in mammalian toxicology and in specific as a tool in assessing compounds for toxicity and safety liabilities in early drug development (Ali et al. 2011; Bambino and Chu 2017; Brannen et al. 2010; Braunbeck 2009; de Esch et al. 2012; Driessen et al. 2013; Fernandes et al. 2017; Guo et al. 2015; Hill 2012; Kari et al. 2007; Nishimura et al. 2015; Scholz 2013; Sipes et al. 2011; Sukardi et al. 2011; Tao and Peng 2009; Ton et al. 2006; Weigt et al. 2011).

In the present study, zebrafish embryos were screened for the hepatotoxic potential of valproic acid (VPA), an antiepileptic drug and known pediatric hepatotoxic agent, as well as 14 selected chemically related substances (analogues; Table 1), by exposing them according to an extended version of OECD TG 236 for 120 h and subsequently evaluating histological changes in liver sections. For comparison between zebrafish embryos and other new approach methods within the EU-ToxRisk project, the total numbers of affected embryos were summarized and an EC_20_ of the liver-altering effect of each compound was calculated.

**Table 1:**
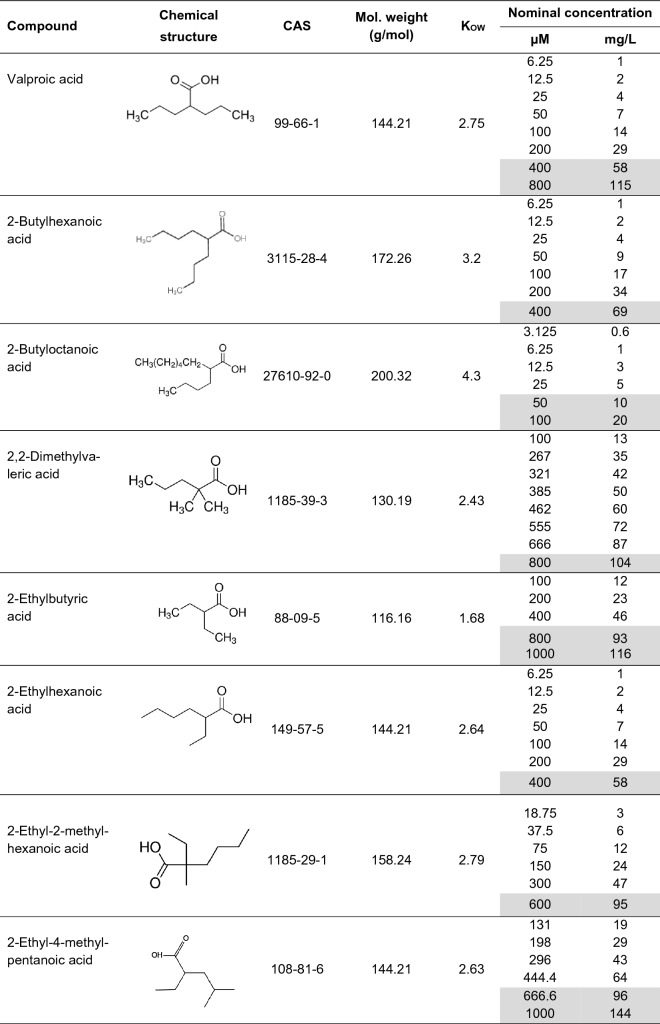

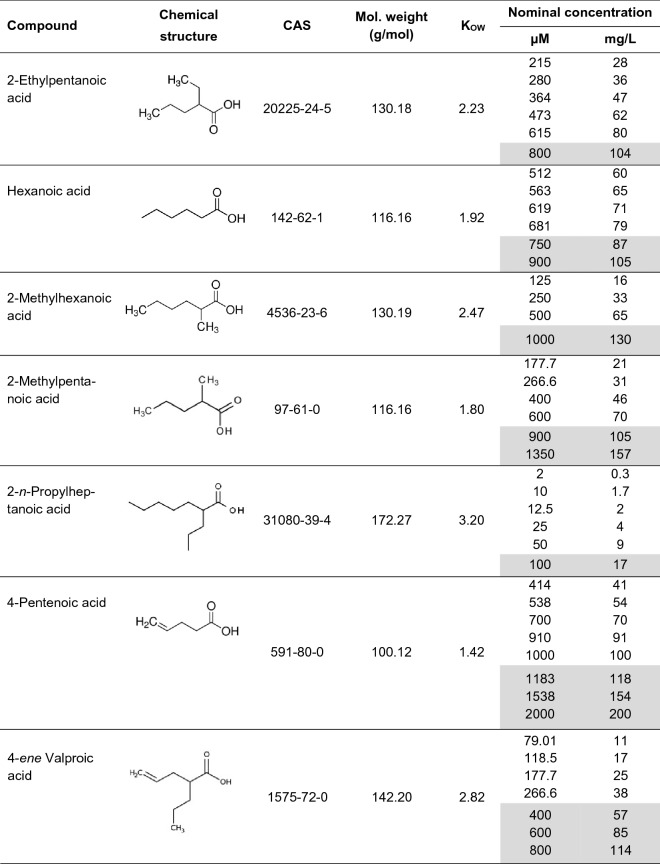
Chemical identity and test concentrations of valproic acid and its analogues tested in the fish embryo acute toxicity test with zebrafish (*Danio rerio*)

Concentrations highlighted in grey caused an early death and non-hatching of treated zebrafish embryos via FET test; these could not be used for histological analysis

Investigations by Herrmann (1993) already indicated a hazard potential of these analogues in humans similar to VPA, and previous FET studies into a smaller set of analogues have shown a wide variety of lethal and sub-lethal effects in the zebrafish embryo (Brotzmann et al. 2020). Although the liver structure of (zebra)fish differs from mammalian systems in several aspects, fundamental developmental processes are well preserved among vertebrates (Tao and Peng 2009), and various studies have even discovered drug metabolic pathways similar to humans, including oxidation, hydroxylation, conjugation, demethylation and deethylation (Vliegenthart et al. 2014).

Briefly, in mammals hepatogenesis starts with the differentiation of hepatoblasts (progenitors of hepatocytes) from the endoderm, which detach from the epithelial layer and form a discrete liver bud. By rapid proliferation, the size of the bud increases (Tao and Peng 2009) and in the next step, hepatoblasts differentiate into functional hepatocytes and bile duct cells (Tao and Peng 2009). In general, studies on mammalian systems, e.g., mice, show high similarities to humans (Vitins et al. 2014); however, mammalian embryogenesis occurs intrauterinely, which makes the embryonic liver inaccessible for continuous studies into processes of liver development. In addition, in mammals the embryonic liver is an early hematopoietic organ; therefore, mutations affecting liver or blood development often cause anemia and even early mortality during embryogenesis, which complicates studies into liver development in mammalian systems (Tao and Peng 2009).

In contrast, during embryogenesis, zebrafish embryos receive nutrients mainly from the yolk (Tao and Peng 2009) and can, therefore, survive and continue to develop normally for a few days even without a functional cardiovascular system (Tao and Peng 2009). In normal zebrafish, development of a physiologically functional liver just takes five days and is thus very rapid when compared to other vertebrate models (Hill 2012). Furthermore, multiple studies have demonstrated that important genes in mammalian hepatogenesis are also mandatory in the development of zebrafish, and fish hepatoblasts originate from the endoderm as they do in humans, mice and other terrestrial vertebrates (Chu and Sadler 2009; Hill 2012; Tao and Peng 2009; Wilkins and Pack 2013). These advantages made the zebrafish embryo a promising model system for studies on general vertebrate liver development and disease (Tao and Peng 2009).

For the present study, valproic acid analogues were selected for three criteria: (1) toxicodynamic properties shared with valproic acid, (2) availability of in vivo endpoint data in mammals and (3) diversity of molecular structure regarding the number and length of side chains. The original aim of the study was a correlation between source compounds, their structural and physicochemical properties and mammalian in vivo endpoint data; however, an in-depth literature research soon revealed a lack of hepatotoxicity data for 10 out of the 15 selected compounds for mammalian test systems and fish (Table 2). In fact, the data available in literature included in vivo developmental data for VPA and some structural analogues in various mammalian models, whereas for zebrafish only VPA data could be localized (Dai et al. 2015; Driessen et al. 2013; Hill 2012; McGrath and Li 2008). This gap was closed with experimental data retrieved from 120 h fish embryo acute toxicity tests (FETs) based on OECD TG 236 (OECD 2013); results for 10 compounds were already presented by Brotzmann et al. (2020), while data for the remaining 5 compounds were generated in the present study.

**Table 2:** In vivo potencies for hepatotoxicity in rats and mice, in specific steatosis, of valproic acid and 14 analogues

In vivo-positive	In vivo-negative	In vivo-unknown
Valproic acid	2-Ethylbutyric acid	2-Methylpentanoic acid
4-*ene* Valproic acid		Hexanoic acid
2-Ethylhexanoic acid		2,2-Dimethylvaleric acid
4-Pentenoic acid		2-Methylhexanoic acid
		2-*n*-Propylheptanoic acid
		2-Butyloctanoic acid
		2-Butylhexanoic acid
		2-Ethylpentanoic acid
		2-Ethyl-2-methylhexanoic acid
		2-Ethyl-4-methylpentanoic acid

According to available mammalian studies, 2-ethylhexanoic acid, 4-*ene* valproic acid and 4-pentenoic acid expressed an in vivo-positive potency in mice and rats for liver toxicity, including indications for steatosis (BG Chemie 2000; Fukami and Williamson 1971; Glasgow and Chase 1975; Juberg et al. 1998; Kassahun and Abbott 1993; Nau and Löscher 1986; Patel and Sanyal 2013; Tang et al. 1995). 2-Ethylbutric acid was also tested in mice and rats, but did not show any hepatotoxic effects, which was classified as in vivo-negative (Api et al. 2020; Di Carlo et al. 1986; Di Carlo 1990). Recordings of liver toxicity in zebrafish embryos were not available for these compounds, although these have been also tested in this model system by Herrmann (1993). Of all test substances, solely VPA provided in vivo-positive data for hepatotoxicity in mice, rats and zebrafish embryos (Abdel-Dayem et al. 2014; Driessen et al. 2013; Espandiari et al. 2008; Ibrahim 2012; Knapp et al. 2008; Löscher et al. 1992; McGrath and Li 2008; Sugimoto et al. 1987; Tong et al. 2005; Willebrords et al. 2015; Zhang et al. 2014).

Given the existing gaps in knowledge for the 15 model substances, the current study was designed to answer the following questions: (1) Which of the compounds show hepatotoxic effects, especially steatosis, in the zebrafish embryo? (2) Is the zebrafish embryo test able to discriminate between chemicals of similar molecular structure? (3) Can the zebrafish embryo be used as an alternative to mammalian model systems for toxicity analysis of analogues?

## Materials and methods

### Chemicals

Except for 4-*ene* valproic acid (Santa Cruz Biotechnology, Dallas, Texas, USA and Carbosynth, Berkshire, UK), 2-butylhexanoic acid (Carbosynth, Berkshire, UK), 2-ethylpentanoic acid and 2-ethyl-4-methylpentanoic acid (Enamine, Kyiv, Ukraine), all test chemicals (Table 1) as well as any other chemical used in the present study were purchased at the highest purity available from Sigma-Aldrich (Dreisenhofen, Germany), unless stated otherwise. Prior to each experiment, test solutions were freshly prepared in artificial water according to Annex 2 of OECD TG 203 (OECD 2019); the pH of the dilution water was adjusted using hydrogen chloride and sodium hydroxide before the addition of the test substance. In fact, all test substances caused a concentration-dependent decrease in pH, in one case up to 6.56. However, since all groups with mortality > 50% were excluded from histological analyses due to a lack of sufficient tissue samples, and since OECD TG 236 allows for a pH range of the test solutions between 6.5 and 8.5, no correction of pH was made.

Known in vivo potencies for hepatotoxicity in mice and rats, in specific steatosis, are summarized in Table 2.

### Fish maintenance

Adult zebrafish (*Danio rerio*) of the wild-type strain ‘Westaquarium’ were obtained from in-house breeding facilities of the Aquatic Ecology and Toxicology Group at the Centre for Organismal Studies (University of Heidelberg; licensed under no. 35-9185.64/BH). Fish maintenance, as well as breeding and spawning conditions were described in detail by Lammer et al. (2009). In brief, a breeding stock of zebrafish aged between 6 and 24 months was used for egg production. Fish was free from externally visible diseases and had not been treated with any pharmaceutical (acute or prophylactic). Females and males were kept together in glass aquaria providing sufficient space for swimming (i.e., ≥ 1 L per fish). Standardized dilution water as specified in ISO 7346–1 and 7346–2 (ISO 1996; 294.0 mg/L CaCl_2_ × 2 H_2_O; 123.3 mg/L MgSO_4_ּ × 7 H_2_O; 63.0 mg/L NaHCO_3_; 5.5 mg/L KCl) or suitable drinking water with ≥ 60% oxygen saturation was used for housing and breeding. Temperature was maintained at 26.0 ± 0.5 °C, and fish was kept under a constant artificial dark/light cycle of 10/14 h. Constant filtering or permanent flow-through conditions guaranteed that ammonia, nitrite, and nitrate were kept below detection limits (0–5, 0.025–1 and 0–140 mg/L, respectively). Fish were fed a commercially available artificial diet (TetraMin™ flakes; Tetra, Melle, Germany) twice daily, occasionally supplemented with *Artemia* nauplii (Sanders Premium Great Salt Lake; Ogden, Utah, USA) or *Paramecium* protozoans of appropriate size, obtained from an own uncontaminated source. Overfeeding was strictly avoided to ensure optimal water quality; remaining food and feces were removed daily.

### Exposure of zebrafish embryos

Exposure was performed according to Brotzmann et al. (2020). Briefly, all test chemicals were tested based on the protocol by the fish embryo acute toxicity (FET) test according to OECD TG 236 (OECD 2013). Only the duration of the experiments was extended to 120 h, which, however, is still within the developmental phase defined as non protected (EU 2010), according to Strähle et al. (2012).

For initiation of each test, freshly fertilized eggs (< 1 h post-fertilization) were seeded into 25 ml crystallizing dishes filled with the respective test solution, and, after control of the fertilization success (fertilization rate and initiation of normal cell division), 10 eggs per test solution were individually transferred into 24-well plates (TPP, Trasadingen, Switzerland) with 1 ml of test solution and 1 embryo per well. All test vessels had been pre-incubated with the test solutions for at least 24 h. During the experiments, embryos were placed in a HettCube 600R incubator (Hettich, Tuttlingen, Germany) at 26.0 ± 1.0 °C under a 10/14 h dark/light regime. The test media were renewed each day (semi-static exposure), and lethal and sublethal effects in the embryos were documented at 24, 48, 72, 96 and 120 h according to OECD TG 236 (OECD 2013) and Nagel (2002), respectively. FETs with a minimum mortality rate of 30% in the positive control (4 mg/L 3,4-dichloroaniline) and a maximum effect rate of 10% in the negative control (dilution water) at 120 h were classified as valid. Nominal test concentrations with a mortality rate of > 50% and non-hatching of embryos were excluded from the analysis, since no samples could be derived for histology. All treatments were tested in three independent runs.

The final (nominal) test concentration range of the test compounds is listed in Table 1. Technically, only 2-*n*-propylheptanoic acid and 2-butyloctanoic acid required the use of dimethylsulfoxide (DMSO; Grüssing, Filsum, Germany) as a solvent; however, for reasons of comparability, all test compounds were dissolved in 100% (v/v) DMSO and then diluted with artificial water to a final nominal test concentration of 0.1% (v/v) DMSO. Test solutions were replaced at 0, 24, 48, 72, 96 h of exposure.

After termination of the treatment at 120 h, the embryos were anesthetized in crushed ice for 30 min and fixed in Davidson’s fluid (220 ml 37% formaldehyde, 115 ml 99% glacial acetic acid, 330 ml 95% ethanol, 335 ml Aqua bidest.) at 4 °C overnight for histopathological examination. One out of three replicates per test substance was used for this screening, while the remaining two replicates were used for toxicokinetic analyses.

### Histopathology

*Fixation, embedding and infiltration* After 24 h fixation with Davidsons’s fluid (Braunbeck et al. 2010), all fish were embedded in separate agarose wells according to the procedure of Sabaliauskas et al. (2006) with moderate modifications: A casting mold made of poly-methyl methacrylate was lined with tape and filled with 800 µl 1% agarose (Life Technologies, Paisley, Scotland) and allowed to solidify at room temperature for 45 min. Fish embryos were rinsed twice in artificial water and positioned horizontally, facing into the same direction in the wells so the spine and both eyes could be seen from above. Wells were filled up with 1% agarose and allowed to solidify for another 45 min. After transfer into embedding cassettes (Roth, Karlsruhe, Germany), the blocks were incubated overnight in 70% ethanol at 4 °C.

The positioned samples were further processed in a semi-enclosed Leica TP1020 benchtop tissue processor (Leica Microsystems, Wetzlar, Germany). In a graded series of ethanol (80%, 90%, 90%, 96%, 96%; 1 h each), isopropyl alcohol (100%; 2 × 1 h) and xylene (100%; 1 h, 12 h and 4 h), samples were dehydrated and infiltrated with Histoplast paraffin wax (Roth, Karlsruhe, Germany; 100%, 2 × 12 h). In a final step, samples were assembled into bigger paraffin blocks with a heated Leica EG 1140 H paraffin embedding module (Leica, Frankfurt am Main, Germany), cooled with a Leica EG 1140 C cold plate (Leica, Frankfurt am Main, Germany) and stored at room temperature until further processing.

*Sectioning and staining* Sections were cut at 4 µm sections with a Reichert-Jung HN 40 microtome (Reichert-Jung, Heidelberg, Germany) and transferred onto microscope slides coated with glycerin albumin (Serva Electrophoresis, Heidelberg, Germany). Hematoxylin–eosin (HE) staining was performed following Mulisch and Welsch (2015): Nuclei and basophilic substances were stained blue by Mayer´s’ hemalum (Roth, Karlsruhe, Germany), whereas cytoplasm, connective tissues and acidophilic substances were stained red with eosin G (Roth, Karlsruhe, Germany). Finally, all slides were coated with X-TRA-Kitt (Medite, Burgdorf, Germany) to prevent oxidation. For all substances, approximately 10 embryos per test concentration were analyzed. Non-hatched embryos were also analyzed, but excluded from the evaluation and calculation of EC_20_ values.

*Histological analysis* Analyses of histological slides were carried out with a Nikon ECLIPSE 90i microscope (Nikon Instruments, Amsterdam, Netherlands) using the Nikon 64-bit software NIS Elements AR 4.00.05. For evaluation, slides displaying the biggest cross sections of the liver were selected, whereas liver sections being divided by the gut were excluded. Embryos displaying any liver alteration such as, e.g., irregular nuclei or reduced hepatocellular diameter were added up for each test concentration, and EC_20_ values of all chemicals were calculated using ToxRat® (vers. 2.10.03; ToxRat® Solutions, Alsdorf, Germany). For measurement of the hepatocellular diameter, 30–40 hepatocytes of each selected cross section of the liver were analyzed using the ImageJ software (Schneider et al. 2012). Mean value and standard deviation per treatment group were calculated with Microsoft Excel.

### Structure–activity relationship (SAR) evaluation

For identification and validation of potential structure–activity relationships, 10 out of 15 compounds were selected for testing and analyzed with respect to histopathology:

Group 1: valproic acid, 4-*ene* valproic acid, 2-ethylhexanoic acid, 2-*n*-propylheptanoic acid, 2-methylhexanoic acid, 2-methylpentanoic acid, 2-ethylbutyric acid, 2,2-dimethylvaleric acid, 4-pentanoic acid, and hexanoic acid.

Based on evident SAR-trends seen for compounds of Group 1 by the arrangement of their EC_20_ values, extrapolations for appropriate test concentration ranges and toxicity potentials of the remaining five valproic acid analogues of Group 2 were made, and additional FETs were carried out without prior range finding:

Group 2: 2-ethyl-4-methylpentanoic acid, 2-ethylpentanoic acid, 2-ethyl-2-methylhexanoic acid, 2-butyloctanoic acid, and 2-butylhexanoic acid.

Thus, in contrast to Group 1 substances, compounds of Group 2 were tested without prior range-finding tests for two reasons: (1) to validate the reliability and predictive power of the observed SAR-trends seen for Group 1 substances and (2) to save test materials for three FET runs, since the available amount of pure compound was limited. In the final step, predictions for Group 2 were validated on the basis of histological observations.

## Results

### Histopathology of Group 1

**Fig. 1:**
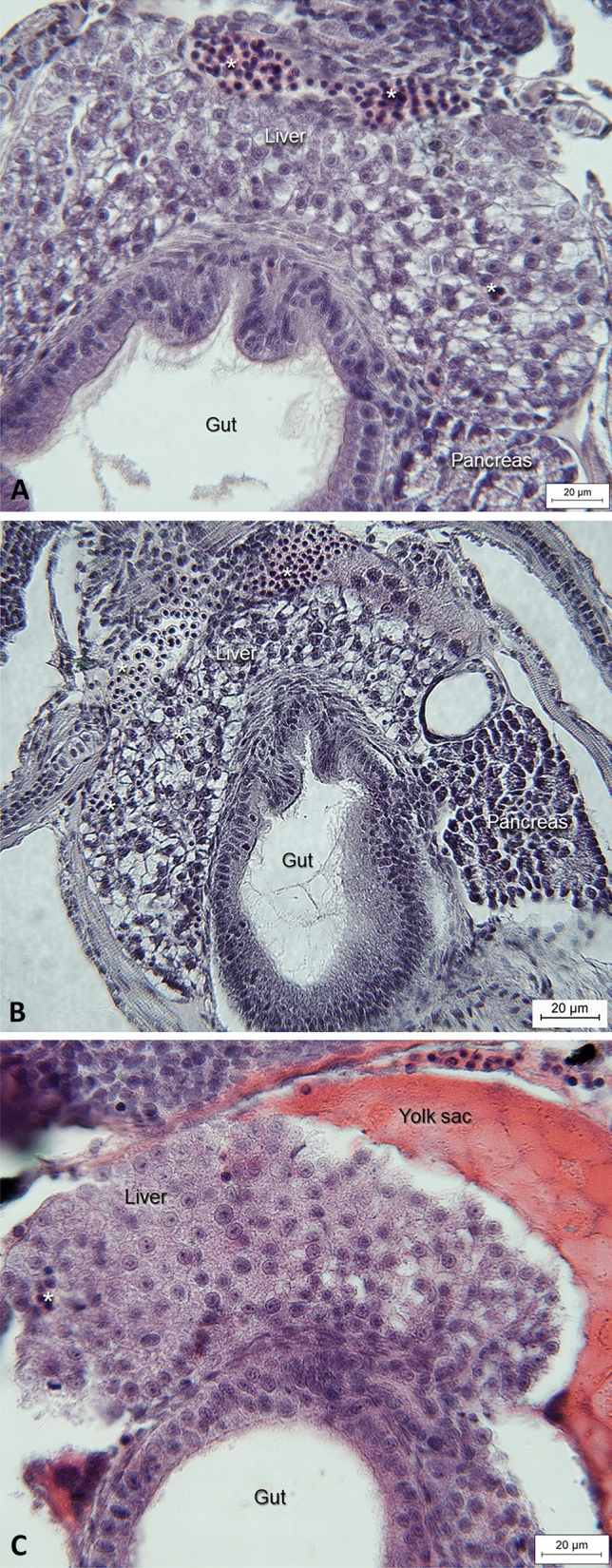
Histological appearance of the liver of zebrafish (*Danio rerio*) embryos at 120 h post-fertilization; hematoxylin–eosin-staining. **a** Negative control, **b** 0.1% dimethyl sulfoxide (solvent control) and **c** 200 µM valproic acid. Evaluation of liver is based on biggest cross sections of the liver per embryo. No differences could be observed between the negative control and the solvent control. Effects of valproic acid (lack of glycogen deposits) could also be observed for all analogues except for hexanoic acid and 4-pentenoic acid. Therefore, valproic acid is representative of the histological appearance of the liver of zebrafish embryos exposed to all positive test substances. *Erythrocytes

Hepatocytes of untreated control zebrafish embryos, as well as embryos treated with 0.1% DMSO were characterized by a regular hepatocellular structure and high amounts of storage materials. Furthermore, in the biggest cross section of the liver per embryo, the organ showed a big diameter reaching from a big left lobe over the midline to a smaller right lobe. While touching the pericardial cavity anteriorly, it grows posteriorly until reaching the head of the pancreas on their right side. In multiple sections, blood vessels containing blood cells were visible. Hepatocytes of untreated and DMSO-treated embryos were big in diameter, 20.3 ± 1.1 µm and 20.6 ± 0.9 µm, respectively, showed regularly shaped nuclei, and a major area of each hepatocyte was characterized by empty spaces formerly occupied by glycogen areas and lipid droplets (both extracted during fixation and/or dehydration procedures; Fig. 1a, b).

Except for hexanoic acid and 4-pentenoic acid, all test substances induced the same histopathological changes: If compared to the negative control and solvent control, the overall size of the livers of treated zebrafish was reduced as the size of individual hepatocytes (up to 43%). Only parts of the livers still contained storage materials, hepatocellular nuclei appeared less regular (Fig. 1c), and blood vessels were only rarely seen. Beside the liver, bigger amounts of yolk were visible on multiple histological slides.

With respect to the severity of the liver alterations, there was a clear-cut concentration–response relationship in the number of affected individuals per treatment group: Whereas VPA, 2-*n*-propylhepatnoic acid, 4-*ene* VPA, 2-ethylhexanoic acid and 2,2-dimethylvaleric acid showed a total percentage of affected (and hatched) embryos of 66–100% at the highest analyzed test concentration with a gradually decreasing percentage at each lower test concentration, 2-ethylbutyric acid, 2-methylhexanoic acid and 2-methylpentanoic acid only expressed a maximum incidence of 11–22% in the highest analyzed test concentration and 0% in the second highest treatment group (Table 3).

**Table 3:** Percentage of zebrafish (*Danio rerio*) embryos per displaying liver alterations after 120 h exposure to valproic acid or its analogues

	Substance	EC_20_ [µM]	Percentage of zebrafish embryos histologically affected per test concentration (120 hpf)							
Group 1	2-*n*-Propylheptanoic acid	10	% of embryos *Test concentration*	100 *50 µM*	100 *25 µM*	50 *12.5 µM*	0 *10 µM*	0 *2 µM*		
	Valproic acid	17	% of embryos *Test concentration*	100 *200 µM*	62.5 *100 µM*	33 *50 µM*	22 *25 µM*	20 *12.5 µM*	10 *6.25 µM*	
	2-Ethylhexanoic acid	28	% of embryos *Test concentration*	**100** ***400 µM***	80 *200 µM*	66 *100 µM*	50 *50 µM*	20 *25 µM*	0 *12.5 µM*	0 *6.25 µM*
	4-*ene* Valproic acid	201	% of embryos *Test concentration*	**100** ***400 µM***	66 *266.6 µM*	9 *177.7 µM*	0 *118.5 µM*	0 *79.01 µM*		
	2,2-Dimethylvaleric acid	378	% of embryos *Test concentration*	**100** ***800 µM***	70 *400 µM*	70 *555.5 µM*	50 *462.9 µM*	20 *385.8 µM*	11 *321.5 µM*	0 *267.9 µM*
	2-Ethylbutyric acid	419	% of embryos *Test concentration*	**100** ***800 µM***	11 *400 µM*	0 *200 µM*	0 *100 µM*			
	2-Methylhexanoic acid	493	% of embryos *Test concentration*	22 *500 µM*	0 *250 µM*	0 *125 µM*				
	2-Methylpentanoic acid	600	% of embryos *Test concentration*	20 *600 µM*	0 *400 µM*	0 *266.6 µM*	0 *177.7 µM*			
Group 2	2-Butyloctanoic acid	2	% of embryos *Test concentration*	80 *25 µM*	57 *12.5 µM*	40 *6.25 µM*	30 *3.125 µM*			
	2-Butylhexanoic acid	4	% of embryos *Test concentration*	100 *200 µM*	0 *100 µM*	33 *50 µM*	60 *25 µM*	60 *12.5 µM*	20 *6.25 µM*	
	2-Ethyl-2-methylhexanoic acid	24	% of embryos *Test concentration*	33 *300 µM*	60 *150 µM*	20 *37.5 µM*				
	2-Ethylpentanoic acid	287	% of embryos *Test concentration*	**100** ***615 µM***	88 *473 µM*	50 *364 µM*	22 *280 µM*	0 *215 µM*		
	2-Ethyl-4-methylpentanoic acid	312	% of embryos *Test concentration*	30 *444 µM*	22 *296 µM*	13 *198 µM*	0 *131 µM*			

Test concentrations highlighted in bold identify non-hatched embryos, which were excluded both from the histological analysis evaluation and the calculation of EC_20_ values. Hexanoic acid and 4-pentenoic acid were excluded from this table, since these analogues did not induce any liver alterations. Order according to the ascending EC_20_ values

### Structure–activity relationship (SAR)

Arranging the analogues by their EC_20_ values (Table 4) in ascending order revealed a correlation between the molecular structure and the liver-altering potency: Non-branched monocarboxylic acids (hexanoic acid and 4-pentenoic acid; framed blue) were inactive, whereas dicarboxylic acids with shorter side chains (2-ethylbutytic acid, 2-methylhexanoic acid and 2-methylpentanoic acid; boxed yellow) as well as tricarboxylic acids with more short than long side chains (2,2-dimethylvaleric acid; framed green) showed a trend towards inactivity. In contrast, dicarboxylic acids with longer side chains (2-*n*-propylheptanoic acid; framed red) showed a strong potency for liver alteration. Symmetry of both side chains also seems to play a role for the potency for hepatotoxicity, since, e.g., VPA and 2-ethylhexanoic acid count the same number of carbon atoms, but express different potencies: the symmetric molecule of VPA is more hepatotoxic than the asymmetric molecular structure of 2-ethylhexanoic acid.

**Table 4:**
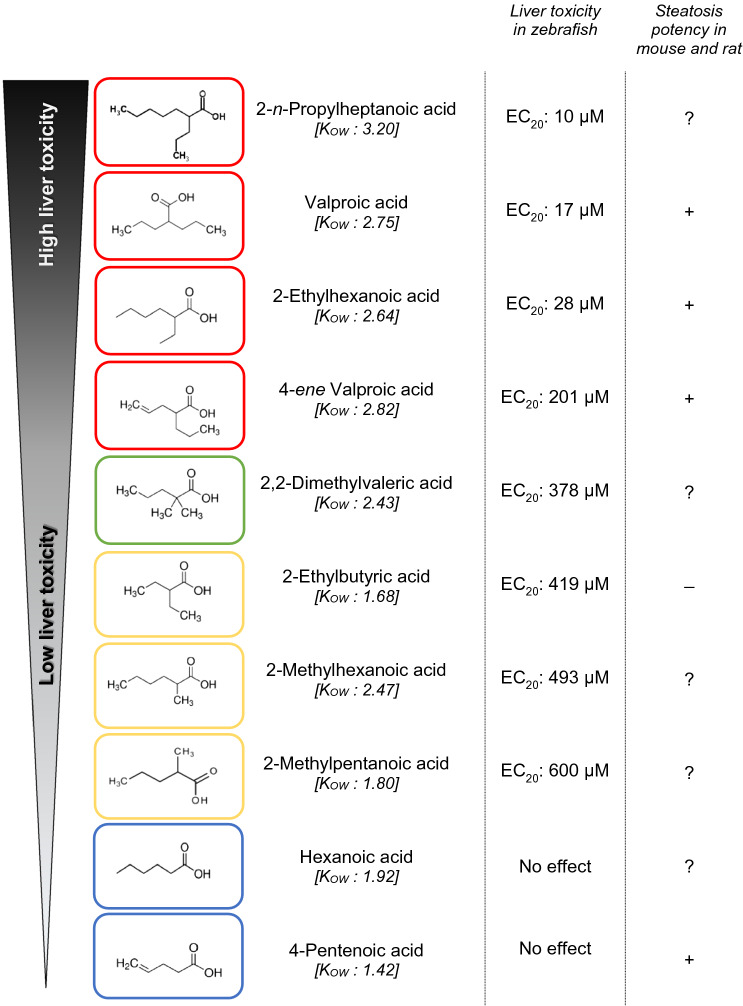
Hepatotoxicity of valproic acid and nine analogues (Group1) arranged according to their EC_20_ values for liver-altering effects in 120 h zebrafish embryos

Results show a correlation between the molecular structure of a substance and its liver-altering potency: Non-branched monocarboxylic acids (framed blue) are inactive; dicarboxylic acids with shorter side chains (framed yellow) and tricarboxylic acids with more short than long side chains (framed green) show a trend towards inactivity; dicarboxylic acids with longer side chains (framed red) show an increased liver-altering potency. + Induction of steatosis in mouse and rat observed; – No induction of steatosis in mouse and rat observed; ? Potency unknown

Interestingly, three out of four chemicals expressing the highest liver-altering potency in the FET (valproic acid, 2-ethylhexanoic acid, 4-*ene* valproic acid) were also in vivo-positive for steatosis in mice and rat (Abdel-Dayem et al. 2014; BG Chemie 2000; Espandiari et al. 2008; Ibrahim 2012; Juberg et al. 1998; Kassahun and Abbott 1993; Knapp et al. 2008; Löscher et al. 1992; Löscher et al. 1993; Patel and Sanyal 2013; Sugimoto et al. 1987; Tang et al. 1995; Tong et al. 2005; Zhang et al. 2014).

Based on this structure–activity relationship, a trend becomes obvious indicating an increased hepatotoxicity with increasing length of the side chain. This observation corresponds with results by Löscher and Nau (1985) investigating the anticonvulsant potency of VPA and some analogues in mice.

### Histopathology of compounds Group 2

For confirmation of the trend observed, predictions for the toxicity of another 5 analogues (substances of Group 2) and their respective test concentration range were made prior to the FET tests (Table 5). Based on their molecular structure, all test compounds were predicted to show a positive hepatotoxic potency in the zebrafish embryo although, similar to 2-ethylbutyric acid and 2-methylhexanoic acid, 2-ethyl-4-methylpentanoic acid was predicted to express a low potency. Position of the additional five analogues is written bold:

**Table 5:**
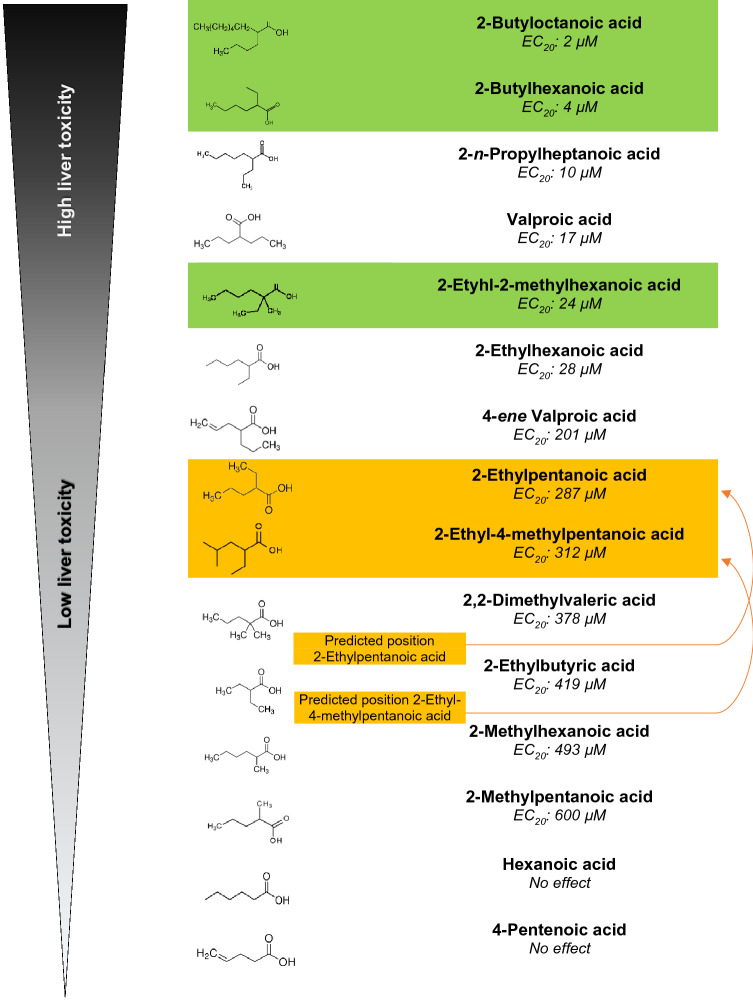
Position of chemicals of Group 2 (highlighted in green and orange) integrated into the trend of substances of Group 1 (cf. Table 4) on the basis of experimental results in the fish embryo test (FET)

Three out of five compounds (2-butylhexanoic acid, 2-butyloctanoic acid and 2-ethyl-2-methylhexanoic acid) were predicted in the correct position for their liver-altering potencies (colored green). Two compounds (2-ethylpentanoic acid and 2-ethyl-4-methylpentanoic acid) had to be corrected by one to two positions (colored orange). Orange arrows = Correction from the predicted potency to the tested liver-altering potency

Prediction for relative toxicity to zebrafish embryos:

**2-Butyloctanoic acid** > **2-butylhexanoic acid** > 2-*n*-propylheptanoic acid > valproic acid > **2-ethyl-2-methylhexanoic acid** > 2-ethylhexanoic acid > 4-*ene* valproic acid > 2,2-dimethylvaleric acid > **2-ethylpentanoic acid** > 2-ethylbutyric acid > **2-ethyl-4-methylpentanoic acid** > 2-methylhexanoic acid > 2-methylpentanoic acid > hexanoic acid = 4-pentenoic acid.

Preliminary FET results confirmed a correct prediction of the effectivity of all test concentration ranges; however, for the exact liver-altering potency, experimental results for two out of the five additional analogues differed from the prediction (underlined; Table 5):

Relative toxicity to zebrafish embryos based on experimental results:

**2-Butyloctanoic acid** > **2-butylhexanoic acid** > 2-*n*-propylheptanoic acid > valproic acid > **2-ethyl-2-methylhexanoic acid** > 2-ethylhexanoic acid > 4-*ene* valproic acid > **2-ethylpentanoic acid** > **2-ethyl-4-methylpentanoic acid** > 2,2-dimethylvaleric acid > 2-ethylbutyric acid > 2-methylhexanoic acid > 2-methylpentanoic acid > hexanoic acid = 4-pentenoic acid.

In effect, all additional analogues of Group 2 proved to be true positives in the zebrafish embryo, and the test concentration range as well as toxicity potency predictions for three substances (2-butyloctanoic acid, 2-butylhexanoic acid, 2-ethyl-2-methylhexanoic acid) were correct. Estimations of two substances (2-ethylpentanoic acid, 2-ethyl-4-methylpentanoic acid) had to be re-adjusted, however, only by one to two positions.

Similar to Group 1, there was also a difference in severity (number of embryos affected) for the analogues of Group 2: 2-Butyloctanoic acid, 2-butylhexanoic acid, 2-ethylpentanoic acid expressed a similar incidence as VPA, i.e., 80–100% at the highest test concentration, and incidences showed a concentration-dependent decline thereafter. 2-Ethyl-4-methylpentanoic acid showed a maximum incidence of 30% in the highest test concentration analyzed; however, instead of a rapid decrease with concentration, 2-methylpentanoic acid, 2-methylhexanoic acid and 2-ethylbutyric acid only displayed a slow percentile decrease at lower test concentrations (Table 3).

## Discussion

### Liver toxicity in the zebrafish embryo

For the correct identification of adverse liver effects in species from different vertebrate classes, three parameters have to be considered: (1) similarities and differences of hepatogenesis, anatomy and morphology of the zebrafish liver relative to mammals, (2) the rapid development of the zebrafish embryo within the first days of its life and, hence, (3) a different morphological state of the liver at each developmental stage. Although knowledge about the morphology and ultrastructure of adult *Danio rerio* liver, which has been described in detail by Braunbeck et al. (1990), Menke et al. (2011), as well as Yao et al. (2012), may contribute to understand the general anatomy and functionality of the liver in fish, additional information is required as a basis for the toxicological evaluation of embryonic hepatocytes.

In mice, hepatogenesis starts at approximately one-third of the way through gestation and is only completed near birth (Chu and Sadler 2009). It starts with the establishment of a population of hepatic precursor cells within the ventral foregut endoderm, which specify into definite hepatoblasts (liver progenitor cells). These precursor cells delaminate from the epithelial layer to form a liver bud, proliferate rapidly and finally differentiate into functional hepatocytes and biliary duct cells (Tao and Peng 2009).

In zebrafish, hepatogenesis is divided into three stages: specification, differentiation and hepatic outgrowth (Chu and Sadler 2009; Hill 2012; Tao and Peng 2009; Wilkins and Pack 2013). During specification, liver progenitor cells originating from the anterior endoderm are identifiable earliest at 16 h and latest between 22 and 24 h by the expression of *hhex* and *prox-1* (Chu and Sadler 2009; Tao and Peng 2009; Wilkins and Pack 2013). Among endoderm-derived organs (i.e., intestine, pancreas, hepatopancreatic ductal system or pneumatic duct and swim bladder), the liver is the organ determined first (Wilkins and Pack 2013). This observation underlines its developmental and evolutionary relevance for the zebrafish embryo, since growth of the whole organism is linked to the exploitation of yolk through the liver.

In the second stage, hepatoblasts aggregate between 24 and 28 h, which leads to the thickening in the intestinal primordium (Hill 2012), and the initiation of differentiation. Molecular markers of mature hepatocytes and biliary epithelial cells are detectable at 32 h (*ceruloplasmin*) and 48 h (*transferrin* and *L-FABP*) (Chu and Sadler 2009; Wilkins and Pack 2013), and CYP-mediated metabolism (be it hepatocellular or extrahepatic) is already active at 36 h (Lörracher and Braunbeck 2020). At 48 h, the liver primordium is clearly discernable as a prominent bud extending from the left of the midline over the yolk (Chu and Sadler 2009), and at approximately 50 h liver tissue is easily recognizable (Hill 2012). At the end of this stage, the liver is located anteriorly between the duct of Cuvier and posteriorly the mid-level of the fin bud (Tao and Peng 2009).

Finally, in the third stage of zebrafish development, the liver changes in size, shape and localization by a rapid proliferation, differentiation and polarization of hepatocytes and the expansion of the biliary system (Chu and Sadler 2009; Wilkins and Pack 2013). This growth phase is initiated approximately at 50 h and continues into the juvenile stage, until the liver parenchyma and the biliary tract are fully developed (Chu and Sadler 2009; Wilkins and Pack 2013).

Between 55 and 72 h, growth of the hepatic vasculature is initiated to facilitate the rapid growth of the organ. Endothelial cells partially encapsulate the liver bud and subsequently start to invade it (Chu and Sadler 2009; Wilkins and Pack 2013). By 72 h, “vascularization is essentially completed, and the liver becomes perfused with blood shortly after” (Hill 2012). At 96 h, the zebrafish liver consists of a larger left lobe that crosses the midline ventral to the esophagus, and forms the smaller right lobe that extends ventrally towards the head of the pancreas. It touches the pericardial cavity anteriorly and overlaps with the anterior portion of residual yolk (Chu and Sadler 2009; Field et al. 2003; Tao and Peng 2009).

At 5 days post fertilization, zebrafish liver embryogenesis is essentially complete, the digestive system is basically functional (Hill 2012), and bile production, serum protein secretion, glycogen storage and lipogenesis are fully operational (Chu and Sadler 2009).

In principle, the developmental stages described in the zebrafish embryo match with hepatogenesis in mammals; however, there are four major differences: (1) For mammalian liver development, hepatic vasculature and hematopoiesis are essential. Mutations of these systems often cause anemia and early lethality, which might lead to complications in the study of liver development (Tao and Peng 2009). This is not the case with zebrafish, since embryonic hematopoiesis does not take place in the zebrafish liver. In fact, zebrafish early liver development is independent of vasculogenesis, which allows the embryo to develop for several days even without cardiovascular circulation (Korzh et al. 2008; Tao and Peng 2009). (2) During the development and differentiation of the hepatic bud in mammals, the septum transversum mesenchyme provides important inductive signals. This structure does not seem to exist in fish; however, the lateral plate mesoderm apparently has an analogous function in zebrafish (Chu and Sadler 2009). (3) The cellular and histological architecture clearly differ between mammals and zebrafish, although these still seem to maintain the same functions, which have already been studied in medaka (*Oryzias latipes*) (Hardman et al. 2007): whereas mammalian livers regularly show portal triads consisting of an artery, a larger vein and a bile duct, teleost fish hepatocytes are more typically organized in plates (hepatocellular cords) lined by sinusoids and biliary ductules, as ramifications of a more irregular biliary tract (Chu and Sadler 2009). (4) In mammals, the biliary system itself consists of extra- and intrahepatic ducts and ductules, whereas in fish preductal epithelial cells are an extra branch on the teleost biliary tree and analogues to the Canal of Hering, which form junctions with canaliculi to collect the bile (Chu and Sadler 2009; Hardman et al. 2007). According to Chu and Sadler (2009), these cells might represent the fish version to hepatic progenitors in other organisms.

Despite these differences, the final general anatomy, organization, cellular composition and function of a healthy adult zebrafish liver are virtually the same as in mammals, and the early embryonic stages of hepatogenesis are similar to that of mice (Hill 2012). Drug metabolization operates similar to human, their metabolic reactions include oxidation, hydroxylation, conjugation, demethylation and deethylation (Lörracher and Braunbeck 2021; Vliegenthart et al. 2014). Likewise, with regard to disease phenotypes, the histopathological syndromes of cholestasis, fatty liver (steatosis) and neoplasia as well as liver regeneration and hepatocarcinogenesis also appear principally comparable in both organisms, even in 5 d old larvae (Amali et al. 2006; Goessling and Sadler 2015; Hill 2012; Spitsbergen et al. 2000), although the processes leading to the phenotypes might be different in detail. Furthermore, extensive research into genetics and tissue cultures uncovered a network of transcription factors and signaling pathways, which are required for forming not only the mammalian liver, but are essential for zebrafish hepatogenesis as well (Chu and Sadler 2009; Tao and Peng 2009; Wilkins and Pack 2013).

Based on the knowledge of these developmental stages, morphology of histological liver sections of non-treated and solvent control embryos becomes reasonable: liver sections stretching from a big left lobe to a smaller right lobe, displaying blood vessels filled with blood cells, as well as multiple regularly shaped nuclei, indicate the proliferation and outgrowth process of a healthy organ at the end of stage three of hepatogenesis in zebrafish (Fig. 1a, b) (Chu and Sadler 2009; Wilkins and Pack 2013).

In contrast, observations made in treated zebrafish embryos indicate numerous symptoms of liver alteration. In the present study, the reduced diameter of hepatocytes was considered as the most important endpoint for the evaluation of histological changes after exposure to valproic acid and its analogues. Other observations included a conspicuous reduction of storage materials, reduced or even missing vascularization (i.e., no or only erratic blood cells between hepatocytes as well as irregular nuclei). The apparent concentration-dependent functional restriction of the liver cells finds its correlate in an overall decline of liver size; this endpoint, however, was ascribed least importance, since the size of an organ can also be linked to the overall size (developmental stage) of the embryo, which has unfortunately not been measured in detail in the present study and could, therefore, not be investigated further (Fig. 1c). Taken together, liver effects recorded in embryos exposed to VPA and its analogues suggest a morphology typical of the end of hepatogenesis stage two. This conclusion could be drawn for all compounds tested within Groups 1 and 2, except for hexanoic acid and 4-pentenoic acid.

There are, however, also controversial observations in previous studies by Passeri et al. (2009), Thakur et al. (2011), as well as Driessen et al. (2013), who described a hepatocellular structure in negative control zebrafish embryos similar to that seen in embryos treated with VPA or its analogues in the present study. This discrepancy is likely due to differences in the fixation procedures: Whereas Passeri et al. (2009), Thakur et al. (2011), as well as Driessen et al. (2013) used only 4% (v/v) paraformaldehyde as fixatives, the present study used a more complex mixture, Davidsons’s fluid, which is known to cause less tissue shrinkage and distortion (Lang 2006; Leimbacher 2009; Simmons and Swanson 2009; Small and Peterson 1982).

Other endpoints such as, e.g., accumulation of vesicular lipid deposits, which might indicate steatosis and were also described by Passeri et al. (2009) and Driessen et al. (2013), could not be confirmed in the present study, since, for an unequivocal evaluation, another fixation and staining method would have been required, namely LipidGreen 2 staining (Chun et al. 2013) or 4% (v/v) paraformaldehyde fixation, followed by PAS-Alcian blue staining (Mulisch and Welsch 2015).

Overall, the liver alterations observed for VPA and 12 of 14 analogues might either be interpreted as a hepatotoxic effect, a retardation or partial inhibition of liver developmental (Cox and Goessling 2015; Farooq et al. 2008). The latter could also be induced indirectly by side effects and might be reversible after termination of the treatment (Raldua et al. 2008). The lack of information about the initiating molecular effects by VPA or its analogues complicates the identification of plausible causes. However, epigenetic experiments revealed that histone deacetylase (HDAC) or DNA methyltransferase activities control both hepatic specification and outgrowth (Chu and Sadler 2009; Farooq et al. 2008). Treating zebrafish embryos with an HDAC inhibitor prior to 24 h reduces *hhex* and *prox-1* expression, resulting in a smaller liver (Chu and Sadler 2009). In specific, *hdac1* and *hdac 3* seem to be involved in patterning and hepatic outgrowth (Chu and Sadler 2009), and, since VPA has been shown to be an HDAC inhibitor in both mammals and zebrafish (Giavini and Menegola 2014; Gurvich et al. 2005; Li et al. 2016; Massa et al. 2005), the liver alterations described might in fact be liver-specific effects and not secondary teratogenic effects.

Moreover, apart from genetic and epigenetic alterations, the pH shift into a slightly acidic milieu can also not be excluded as a trigger for the effects observed. Although there are no reports in literature on pH-dependent changes in zebrafish liver architecture and zebrafish embryos are regarded to be fairly tolerant to pH variations between pH 6.5 and 8.5 (OECD 2013), it should still be noted that pH may profoundly affect the specification and solubility of the test solutions, thus changing the availability of the compounds to zebrafish embryos.

### Discrimination of molecular similarity of analogues by a structure–activity relationship

Although the histopathological observations can per se neither categorize a definite in vivo-positive or negative potency for liver toxicity nor identify steatosis, calculations and subsequent analyses of EC_20_ values for liver-altering effects clearly allowed to correlate a decrease of hepatotoxic activity with decreasing side chain length.

This observation corroborates similar conclusions by Herrmann (1993); however, the length of side chains is not the sole determinant for the hepatotoxic potency, since the number of side chains apparently also plays an important role. Compounds with one side chain were non-toxic; substances with three side chains, namely two short and one long side chain, were less toxic than those with two side chains. Furthermore, symmetry of the side chains seemed to be another important parameter, as was evident for, e.g., VPA and 2-ethylhexanoic acid: Both compounds have the same number of carbon atoms; however, while both side chains of VPA are equally long, 2-ethylhexanoic carries a longer and shorter side chain, which decreases its toxicity potential. This rule could be confirmed for the comparison of 2-ethylbutyric acid and 2-methylpentanoic acid (Table 4) and was also observed in two human cell lines, namely HepG2 and HepaRG (Escher et al. 2022). Finally, compounds with higher K_OW_ had lower EC_20_ values and showed higher hepatotoxic potencies (Table 4); hence, compounds with high lipophilicity seem to be better accessible by the zebrafish embryo, which can also lead to elevated accumulation within the organism (de Koning et al. 2015). An additional parameter influencing the absorption of substances is pH-dependency of acids such as those tested in the present study. Although zebrafish embryos are quite tolerant to pH variations between pH 6.5 and 8.5 (OECD 2013), pH may certainly affect both speciation and solubility of the test compounds by manipulating the ratios between ionized and non-ionized molecules and, thus, manipulating the availability of the compounds to the embryos. In case of pH adjustment, the observed SAR-trend would become more distinguished due to differential absorption capacities and low activity analogues would even have needed relatively higher (nominal) test concentrations for inducing hepatotoxic effects at all, thus confirming the current conclusions. For confirmation, a comparison of bioavailability in pH-adjusted *versus* non-adjusted test scenarios is underway.

As a consequence, results suggested a high potency for liver-altering effects for dicarboxylic acids with long side chains, namely 2-*n*-propylheptanoic acid, valproic acid, 2-ethylhexanoic acid and 4-*ene* valproic acid. In contrast, monocarboxylic acids (hexanoic acid and 4-pentenoic acid) did not show any alterations even at the highest concentrations tested. Interestingly, three out of four chemicals expressing the highest potency liver alteration in the FET (valproic acid, 2-ethylhexanoic acid, 4-*ene* valproic acid) were also in vivo-positive in mice and rats with respect to the development of steatosis (Abdel-Dayem et al. 2014; BG Chemie 2000; Espandiari et al. 2008; Ibrahim 2012; Juberg et al. 1998; Kassahun and Abbott 1993; Knapp et al. 2008; Löscher et al. 1992; Patel and Sanyal 2013; Sugimoto et al. 1987; Tang et al. 1995; Tong et al. 2005; Willebrords et al. 2015; Zhang et al. 2014).

This structure–activity relationship strongly correlates with conclusions drawn from studies with *Candida tropicalis* (Bell 1971), mosquito larvae (*Culex pipiens quinquefasciatus*) (Hwang et al. 1974; Ikeshoji and Mulla 1974), *Xenopus laevis* embryos (Dawson et al. 1996), rats (Ambroso et al. 1999; Hisaki et al. 2020), human HepG2 and HepaRG cells (Escher et al. 2022) and studies by Löscher and Nau (1985), who investigated the anticonvulsant potency of VPA and some analogues in mice. According to Löscher and Nau (1985), analogues with shorter side chains are weakly active as anticonvulsants, while longer side chains increase the potency, but also the sedative and hypnotic activities, as well as the teratogenic toxicity. Non-branched monocarboxylic acids and cyclic compounds expressed weak or no activity, while addition of a methyl group in position 1 at a ring of, e.g., cyclohexanoic acid increased the anticonvulsant potency without altering LD_50_ values, and an additional branch with methyl group at C2 enhanced the anticonvulsant potency considerably (Löscher and Nau 1985).

In subsequent studies, Nau et al. (1991) even manifested structural pre-requisites for the expression of significant exencephaly formation in mice, namely the connection of a tetrahedral α-carbon atom to a free carboxyl function, a hydrogen atom and two alkyl groups. As a result, in mice the maximal teratogenic potency is found, if the two alkyl chains branch on C2 and contain exactly 3 carbon atoms each, as it is shown by e.g., VPA. Elongated or shortened carbon chains reduced the activity; however, in zebrafish only shorter side chains reduced the teratogenic potential. Nau et al. (1991) also observed an enhanced potency reduction for shortened alkyl chains (e.g., 2-ethylpentanoic acid) rather than extended chains, e.g., 2-*n*-propylhexanoic acid, and elongation of just one side chain did also not increase the anticonvulsant potency (Löscher and Nau 1985). This could be confirmed by 2,2-dimethylvaleric acid in the present study regarding both its molecular structure (one long and two short chains) and its low hepatotoxic potency.

Most interestingly, studies by Nau and Löscher (1986), Hauck and Nau (1989a), Hauck and Nau (1989b) as well as Hauck et al. (1990) discovered that the induction of a double bond between C2 and C3 (e.g., e-2-en-VPA) or between C3 and C4 (e.g., 3-en-VPA) abolished teratogenic activity in mice, while introduction of a double bond (e.g., (+) 4-en-VPA) or a triple bond (e.g. (+)-4-yn-VPA) at C4 resulted in substances with high teratogenic activities in mice. In the present study, only 4-*ene* VPA and 4-pentenoic acid contained a double bond; while 4-pentenoic acid did not show any liver-altering effects, 4-*ene* VPA actually expressed a higher liver-altering potency than other compounds. However, focusing on its EC_20_ value, the concentration gap to 2-ethylhexanoic acid became apparent: to be specific, 28 µM for 2-ethylhexanoic acid and 201 µM of 4-*ene* VPA (Table 4). In a QSAR analysis based on mice data obtained by Löscher and Nau (1985), Bello-Ramírez et al. (2002) observed that double bonds at either side chain enhanced the lipophilic character of a compound and facilitated crossing of the blood–brain barrier. Such substances showed a higher anticonvulsant potency and higher reactive character, but also a higher metabolic activity and reduced stability. Based on this information, the conspicuous EC_20_ reduction of 4-*ene* VPA relative to 2-ethylhexanoic acid might be explained by its reduced stability in the zebrafish embryo or by diverse metabolic properties of this model system.

To verify the reliability of the structure–activity relationship (SAR) set up for compounds of Group 1, the hepatotoxic potencies of additional compounds (Group 2) were predicted by venturing (1) a prediction of the test concentration ranges in the FET and (2) the exact position within Group 1 for liver-altering effects (Table 4) *before* conducting the tests. FET results showed a success of 100% for predicting the test range; however, for exact EC_20_ values, minor corrections had to be made for two compounds, namely 2-ethylpentanoic acid and 2-ethyl-4-methylpentanoic acid (Table 5). Yet, corrections in positions were minor, and prognoses based on trends seen for Group 1 compounds in the structure–activity relationship were roughly correct (Table 5). Thus, the present study was able to confirm that compounds with similar molecular structure can be discriminated not only on basis of mammalian data, but also based on zebrafish embryo data.

### The zebrafish embryo as an alternative to mammalian test systems?

Overall, the data obtained document a concordance of SAR-effects between mice, two human cell lines (HepG2 and HepaRG) and zebrafish embryos (Escher et al. 2022). Therefore, the present study supports the use of *Danio rerio* embryos for testing chemicals of similar molecular structure for their hepatotoxic potency just like the test systems mentioned above.

However, there are limitations: Although test results align in 100% with the estimations for test concentration ranges, and therefore, confirmed all chemicals of Group 2 to be true positives in the zebrafish embryo, predictions of the exact liver-altering potency had to be adjusted for two out of five compounds (Table 5). Furthermore, only one set and type of compounds was tested; thus, the applicability of this structure–activity relationship to other chemicals needs to be confirmed. Since experimental data are only available for four model organisms so far, the transferability to other model systems also remains to be analyzed.

An improvement of the predictive power of the SAR might be reached by inclusion of further parameters such as effect severity or K_OW_. An analysis of effect rates and hatching success showed strongest liver alterations in 100% of embryos treated with the highest test concentrations of 2-butylhexanoic acid (200 µM), 2-*n*-propylheptanoic acid (50 µM) and VPA (200 µM). These three analogues also ranged among the four test compounds with the highest K_OW_ (3.2, 3.2 and 2.75 respectively; Tables 1, 4). In contrast, analogues with a relatively low effectivity (11–22% of individuals affected) at the highest test concentration also showed relatively low K_OW_ values (2-methylhexanoic acid (500 µM; K_OW_: 2.47), 2-methylpentanoic acid (600 µM; K_OW_: 1.80) and 2-ethylbutyric acid (400 µM; K_OW_: 1.68)). With decreasing concentrations, substances characterized by a high K_OW_ and high liver toxic potency expressed a gradual decrease in the number of affected embryos, whereas compounds with low K_OW_ and low liver toxic potency resulted in a complete lack of embryos affected (Table 4). Thus, quantification of effect severity with respect to the potency for liver alterations in hatched embryos may already allow for a clear identification of an in vivo-positive and negative read-out not only in fish, but also in mammals (and humans). In the present study, 2-*n*-propylheptanoic acid, VPA, 2-ethylhexnoic acid, 4-*ene* VPA, 2,2-dimethylvaleric acid as well as all analogues of Group 2 would be considered as in vivo-positive, while 2-ethylbutyric acid, 2-methylhexanoic acid and 2-methylpentanoic acid would be predicted as in vivo-negative. Concordance between known in vivo mice and rat data (Table 2), and zebrafish embryo results would thus be 80%. Further adjustment of the SAR might even further improve its predictive power and enhance its applicability development for other chemicals.

## Conclusions

Results of the present study clearly demonstrate a correlation between the molecular structure and the hepatotoxic potency of VPA and 14 of its analogues in zebrafish embryos, which can be documented in a structure–activity relationship (SAR). Initially deduced by FET and histopathological data of VPA and 9 analogues, the observed SAR was successfully verified for a second set of five VPA analogues. Predictions made for effective test concentration ranges of analogues in Group 2 were 100% correct, whereas the prognosis of the relative liver-altering potency had to be marginally adjusted for two compounds. Implementation of effect severity and K_OW_ might help to improve the SAR as a tool for teratogenicity evaluation of new chemicals.

From a developmental point of view, the zebrafish embryo showed strong potential as a model in vertebrate liver development including a good correlation with mammals (Hill 2012). Hence, this model might provide an excellent tool to bridge the gap between subcellular and cell-based systems and relevant vertebrate models. As a result, knowledge gained from such studies might contribute to a better understanding of the molecular and genetic mechanisms underlying developmental processes not only in fish, but also in other vertebrate classes, including humans as postulated by Tao and Peng (2009).
